# Not Just Sitting By: Family Caregivers’ Perspectives on Triadic Communication

**DOI:** 10.1093/geroni/igaa057.1892

**Published:** 2020-12-16

**Authors:** Karla Washington

**Affiliations:** University of Missouri School of Medicine, Columbia, Missouri, United States

## Abstract

Most studies of communication in gerontology and palliative care focus on dyadic communication. In reality, encounters in palliative oncology very often involve three or more people: a healthcare professional, a patient, and a family caregiver. This triadic communication differs in important ways from communication involving only two parties. In this secondary analysis of qualitative data collected during a randomized controlled trial of a psychosocial intervention for family caregivers receiving palliative oncology services, researchers explored family caregivers’ (n = 63) perspectives on triadic communication encounters involving themselves, the patient and one or more clinicians. Family caregivers tended to appreciate clinicians’ efforts to involve them in communication, rather than regarding them as “just sitting by” the patient. Many perceived that their own wishes regarding information provision were often ignored and reported that their own coping and wellbeing were not often assessed outside of encounters with the specialist palliative care team.

